# The emergence of disease‐preventing bacteria within the plant microbiota

**DOI:** 10.1111/1462-2920.15896

**Published:** 2022-01-12

**Authors:** Tomislav Cernava, Gabriele Berg

**Affiliations:** ^1^ Institute of Environmental Biotechnology Graz University of Technology Graz 8010 Austria; ^2^ Leibniz‐Institute for Agricultural Engineering Potsdam Potsdam 14469 Germany

## Abstract

Microbiome studies have facilitated the discovery of harmful as well as beneficial microorganisms over the last years. Recently, distinct bacteria were found within the microbiota of crop plants that confer disease resistance to their hosts. Although it is well known that the interplay between microbes and plants can result in improved plant health, the phenomenon of holistically disease‐preventing bacteria is new. Here, we put the recent discoveries of disease‐preventing bacteria in context with decade‐long plant microbiome research that has preceded them. In addition, we provide explanations as to why disease resistance in certain plants, mediated by specific bacteria, has only recently been discovered. We argue that such findings were primarily limited by technological constraints and that analogous findings are very likely to be made with other plant species. The general concept may even be extendable to additional groups of organisms. We, therefore, suggest the introduction of the specific term *soterobiont* in order to facilitate an unambiguous definition of disease‐preventing microorganisms within the microbiota of higher organisms.

## Introduction

Throughout its history, humankind has been struck by devastating disease outbreaks that have affected us either directly or through our food base. Our ambitions to prevent such catastrophic events in the future, by controlling human, animal and plant pathogens, have immensely profited from the technological advancements of the past decades. Microbiome research currently provides one of the most versatile approaches to address infectious diseases that have remained unexplained and elusive as of now (Cho and Blaser, 2012). By analyzing microbial communities that are found in contrasting host phenotypes, various emerging diseases were already linked to distinct pathobionts; the term describes a wide range of potentially disease‐causing organisms, which under specific genetic or environmental conditions can occur as a harmless symbiont in their respective hosts (Jochum and Stecher, 2020). It is noteworthy that the term ‘pathobiont’ is still under discussion, especially with regard to its universal applicability to describe a broad spectrum of microorganisms with a distinct pathogenic potential within a specified microbiota (Jochum and Stecher, 2020). Various members of enterobacteria commonly found in mammalian intestines constitute the most prominent examples for known pathobionts that were discovered in the frame of microbiome studies (Kitamoto *et al*., 2020). In one specific example, it was shown that a particular strain of *Escherichia coli* in the intestine can use metabolic reprogramming to outcompete other bacteria under favourable conditions and cause harm to its host (Kitamoto *et al*., 2020). While the discovery of pathobionts provided valuable clues for the understanding of so‐far unexplainable disease emergence, less is known about potential health effects associated with beneficial members of the microbiota.

In populations of higher organisms, it is often observed that certain individuals are resistant to particular pathogens that are harmful or even deadly to other individuals of the same population (Goujon *et al*., 2013). This phenomenon can be often explained by genetic predisposition or by the involvement of their innate or acquired immune system; however, in most cases the reasons for the observed resistances remain elusive when only host genetics are considered (Matsumoto *et al*., 2021). In many plant species, disease resistance is commonly observed for individual plants as well as for whole populations in a defined geographic region (Laine *et al*., 2011). Disease‐suppressive soils remain a well‐documented but still mostly unexplained phenomenon, where specific, Gram‐negative bacteria are assumed to play a key role in maintaining a disease‐free environment for plant cultivation (Schroth and Hancock, 1982; Schlatter *et al*., 2017). In many other observations of increased disease resistance in plants, it was found that microorganisms can be involved in conferring systemic resistance by triggering various immune responses (Pieterse *et al*., 2014). In contrast to genotype‐specific disease resistance that is commonly achieved via breeding and mostly specific for a distinct plant–pathogen combination, systemic resistance can include a wide range of inducers and structurally unrelated compounds as effectors. The underlying mechanisms are complex and mostly based on the recognition of molecular patterns of either harmful or beneficial microbes. It was also found that certain members of the native plant microbiota, such as various commonly occurring pseudomonads, can make use of this widespread mechanism in the rhizosphere; however, they were not shown to confer holistic disease resistance to their hosts so far. During the last decades, plant microbiome research has mostly focused on the so‐called rhizosphere, which describes soil surrounding plant roots that is influenced by plant metabolites; the rhizosphere can attract and enrich beneficial microorganisms (de Vries *et al*., 2020). This plant‐associated habitat and its beneficial microorganisms are known for more than 100 years; the rhizosphere was described in 1904 by Lorenz Hiltner, and its implications for plant functioning were gradually found to go further than improving disease resistance and better adaptability to adverse environmental factors (de Vries *et al*., 2020). Endophytes, which are another important group of plant‐associated microorganisms, designate specific organisms, which reside inside plant tissues and that can confer extensive adaptation capabilities to their hosts (Márquez *et al*., 2007). Although they generally occur at substantially lower numbers than rhizosphere microbes, they are known to engage in more intimate interactions with their hosts; especially due to their localization which facilitates an extensive exchange and interplay of metabolites.

## The emergence of disease‐preventing bacteria in the plant microbiota

Only recently, two independent studies have confirmed the presence of distinct microorganisms in the plant microbiota that can shape their host phenotypes by evoking disease resistance against bacteria and fungi that corresponds to that of a disease‐resistant plant genotype (Carrión *et al*., 2019; Matsumoto *et al*., 2021). These microorganisms have disease‐preventing properties in contrast to well‐known pathobionts; they prevent the outbreaks of distinct fungal and bacterial diseases whenever they occur in sufficient density. Moreover, their disease protection is holistic by the means of being indistinguishable from host immunity, which differentiates them from all previously discovered, disease‐resistance‐improving microbes. In the past, many beneficial microorganisms have been described that can only provide partial protection against disease in a plant population. Recently, however, in sugar beets (*Beta vulgaris* L.), two endophytes that naturally occur inside plant roots, and were later assigned to the bacterial genera *Chitinophaga* and *Flavobacterium*, were shown to confer resistance to the host plant against *Rhizoctonia solani* that causes damping‐off disease (Carrión *et al*., 2019). In rice (*Oryza sativa* L.), a seed‐endophytic *Sphingomonas melonis* strain was shown to confer disease resistance against *Burkholderia plantarii* that causes seedling blight (Matsumoto *et al*., 2021). The seed endophyte was shown to be naturally transmitted across four successive plant generations and to be transmittable to various disease‐susceptible *O*. *sativa* japonica as well as indica varieties where it evoked a disease‐resistant phenotype. In terms of the specific mode of action of the identified microorganisms, all of them were shown to produce secondary metabolites, such as phenazines, polyketides or specific organic acids, which are highly efficient against the respective plant pathogens (Carrión *et al*., 2019; Matsumoto *et al*., 2021). Moreover, all of the identified disease‐preventing bacteria have in common that they are plant endophytes and not rhizosphere bacteria, which were explored in much more detail during the last decades. The well‐known, intimate association between endophytic microbes and plants might be the key for their success and the same time the reason why they have remained undiscovered until recently. Microbiome analyses targeting the plant endosphere are more complex and hampered by the excessive presence of host tissues in typical samples. Advances in sequencing techniques as well as the development of specific pipelines for bioinformatics processing have contributed to a better understanding of this important microenvironment in recent years.

## What can we expect in the future in terms of disease‐preventing bacteria discovery?

We assume that many more plant‐associated bacteria with disease‐preventing traits will be discovered in the future. In the most optimistic scenario, we can expect the existence of at least one disease‐preventing bacterium, or a different microorganism (fungi and archaea are also promising candidates), for every infectious disease. It will likely take decades before we know with sufficient certainty the frequency with which they occur in nature and if they constitute specifically co‐evolved, complementary mechanisms of disease protection in distinct host organisms. In the near future, their discovery can be facilitated if microbiome researchers implement the general hypothesis that a disease‐preventing microorganism might be involved whenever disease‐resistant phenotypes within an otherwise disease‐susceptible population are not attributable to (i) acquired immunity, (ii) innate immunity and (iii) other variations in host genetics; including epigenetics. Currently, there are several challenges that impede the discovery of disease‐preventive microorganisms. We argue that they are particularly of technical nature, especially with regard to the limitations that still exist in the generation and processing of microbiome data. Discovery of disease‐preventing microorganisms will require targeted correlation analyses in most cases. However, microbiome‐wide correlation analyses still mostly rely on short sequence fragments of distinct marker genes, which limit the resolution of potential discoveries (Berg *et al*., 2020). Certain disease‐preventing microbes might only be distinguishable at strain level from microbes without this trait that belong to the same species. They can only be discovered if below‐species‐level microbiome analyses are implemented; the reconstruction of genomes from metagenomic data (metagenome‐assembled genomes) provides a viable strategy for such approaches, but the financial costs for the required datasets are still a major limiting factor. The analysis of endophytes is hampered by an additional circumstance; due to their localization inside host tissues, the analysis of their genetic information or other molecular building blocks (e.g. proteins and lipids) is hampered by the excess of host tissues that are found as prevalent contaminants in such analyses. Currently, there are specific solutions available for the analysis of genetic markers in ‘host‐rich’ samples; however, they are not applicable for the abovementioned below‐species‐level microbiome analyses, which thus must still rely on sheer sequencing depth. Another important barrier that especially affects endophyte studies is the low proportion of their cultivable representatives (Lagier *et al*., 2018). Further developments in so‐called ‘culturomics’ techniques that focus on the cultivation of yet‐uncultivable microorganisms can contribute to a higher availability of sufficiently characterized endophytes. In addition to technological constraints, there is a general lack of targeted screening strategies aimed at discovering disease‐preventing microorganisms. Future discoveries of such microorganisms will be facilitated if their potential presence in disease‐resistant plants is acknowledged. For this purpose, an unambiguous term for such microorganisms may stimulate the development of upcoming screening strategies.

## Introduction of the term *soterobiont* for disease‐preventing microorganisms

**Fig. 1:**
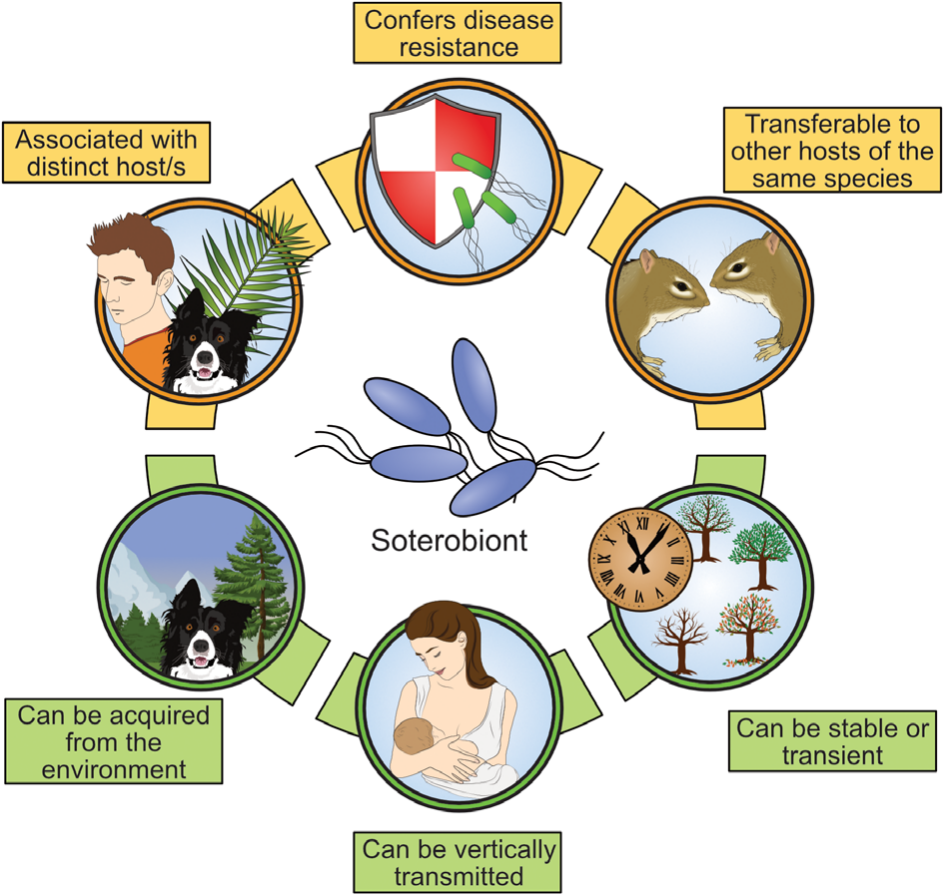
Essential and ancillary traits of a *soterobiont* and known transfer mechanisms between hosts. Traits that are essential for an organism to be termed a *soterobiont* include the association with at least one defined host organism, immune‐system‐like protection against one or multiple specified diseases and transferability to confer disease resistance to disease‐susceptible individuals of the same species (visualized in green panels). Ancillary traits that vary among *soterobionts* include the route of their acquisition by the host (environment or vertical transmission) and the duration of the association (stable or transient; visualized in orange panels). The visualized traits are not exhaustive and are likely to be further refined after more disease‐preventing microbes are characterized.

We argue that a specific designation for disease‐preventing microorganisms could draw further attention in this direction and result in more targeted approaches for their identification in the future. We, therefore, suggest the introduction of the specific term *soterobiont* (plural: *soterobionts*) in order to facilitate an unambiguous definition of disease‐preventing microorganisms in the plant microbiota and potentially beyond. It derives from the ancient Greek goddess Soteria (Ancient Greek: ∑ωτηρία), which was believed to provide safety and deliverance from harm (Suk and Jim, 2017) and ‘‐biont’ which describes a discrete living organism as also found in symbiont and pathobiont (Jochum and Stecher, 2020). From the semantic point of view, *soterobiont* should be understood as one specific microorganism, while the plural form *soterobionts* refers to several or a group of microorganisms. Similar to pathobionts, further research efforts will be needed to refine the usage of the term and further characterize their essential properties, as well as to determine whether the term is universally applicable to microorganisms with a potential to confer disease resistance. According to current knowledge, *soterobionts* can act either as an individual species (Matsumoto *et al*., 2021) or as a defined group of different species (Carrión *et al*., 2019) as shown in the frame of two recent discoveries. They should not be confused with other plant‐associated microorganisms that can reduce disease incidence without evoking an unambiguously resistant host phenotype. The three essential features of a *soterobiont* include prevention of one or multiple diseases, an evident association with at least one defined host organism, and its transferability to confer disease resistance to disease‐susceptible individuals of the same species (Fig. 1). It can be assumed that *soterobionts* can be acquired from the environment, especially from soil in terms of plant populations, or that they can be maintained within a population via vertical transmission to the offspring (Matsumoto *et al*., 2021). We suggest that a *soterobiont* must fulfil the experimentally verifiable requirement to confer disease resistance to an otherwise disease‐susceptible host of the same species, which is in analogy to Koch's postulates for the definition of a pathogen. In contrast to a pathogen's ability to infect a healthy host, a *soterobiont* reproducibly confers disease resistance in a process that is indistinguishable from an innate or acquired immune response. While isolation of the disease‐causing pathogen is traditionally required to fulfil Koch's postulates, we suggest that a *soterobiont* can also be verified, solely based on unambiguous genetic information, i.e. its whole genome must be recovered and it must be proven that solely the presence or absence of an organism with the specified genetic information results in a resistant or susceptible phenotype. A cultivation‐independent validation of *soterobionts* will be required if an organism can only be transferred by the means of a direct transplantation from one host to another to evoke a desirable effect in its recipient. *Soterobionts* that can be isolated and cultured should be nevertheless subjected to whole‐genome sequencing in order to allow clear differentiation between them and related, non‐soterobiont species and strains. In case that host immune responses are triggered by a pathogen attack, it has to be clearly shown that they would not provide sufficient protection (non‐resistant phenotype) in absence of the *soterobiont*. Whenever there may be doubts on a *soterobiont's* efficacy in conferring disease resistance, immunodeficient host models should be employed to confirm the implications of a potential candidate organism. All the above‐mentioned traits provide a rough framework for the characterization of *soterbionts*; it will certainly require further refinement after the discovery of more disease‐preventing microorganisms in the future.

## Concluding remarks

The discovery of disease‐preventing microorganisms could provide the basis for new approaches to address prevalent diseases in plants, animals and humans. Although *soterobionts* were only recently described, they might be widespread constituents of the microbiota and could explain certain disease resistance in individuals or populations that was previously unknown. It is imperative that their occurrence in the microbiota of higher organisms be further explored in order to harness their potential for disease prevention and development of therapeutic agents. Future discoveries will not only rely on technological developments, but primarily on hypothesis‐driven microbiome analyses of healthy and diseased individuals where *soterobionts* may be involved.
